# Study on Fatigue Allowance Formulation Based on Physiological Measurements

**DOI:** 10.3390/s24227393

**Published:** 2024-11-20

**Authors:** Li Qu, Juntong Zhang, Di Wang, Lin Zhang, Zhunan Wu

**Affiliations:** School of Management Science and Engineering, Beijing Information Science and Technology University, Beijing 100192, China; quli@bistu.edu.cn (L.Q.); pandadi@126.com (D.W.); zhanglin@bistu.edu.cn (L.Z.); nancywu.ja@163.com (Z.W.)

**Keywords:** fatigue allowance, physiological measurement, mechanical processing, continuous operation, simulation experiment

## Abstract

The fatigue allowance effectively mitigates declines in physiological state due to work fatigue. An appropriate allowance rate facilitates timely recovery for employees and serves as a crucial basis for labor quota formulation. In this paper, the action mode in mechanical processing was extracted and disassembled into six action units. The study conducted fatigue measurement experiments based on physiological measurement methods, including exercise fatigue tests at different frequencies and work fatigue tests over varying durations. As the frequency of actions increased, the rate monotonic scheduling index showed a linear increasing trend and the degree of fatigue caused by the action was different. The fatigue coefficient of different action units and the fatigue index of the fatigue instability period were obtained by fitting. Hazard ratio indicators showed significant differences, and the corresponding fatigue recovery rest time was obtained for different continuous operation hours. By further fitting the above data, a fatigue relaxation rate model suitable for simulating operation methods was obtained (the fatigue coefficient for the simulated operations in this study is 0.076152) which could provide a reasonable basis for the formulation of fatigue allowance rates for machining methods.

## 1. Introduction

The production processes of enterprises are not continuous, as workers frequently interrupt and stop their tasks due to various factors. Therefore, when establishing labor quotas, it is essential for enterprises to include appropriate work allowance time to compensate employees adequately. However, enterprises often neglect the careful formulation of allowance time when setting labor quotas. The national standard ‘The Time Measurement Method of Labor Quota’ (GB/T 23859-2009) [1] provides detailed elaboration on the application scope, methods, and requirements for labor quotas. However, it lacks a unified standard for fatigue allowance. This absence may hinder the development of harmonious labor relations and could even lead to production safety accidents due to the accumulation of work fatigue. There is no uniform definition of work fatigue [2], but it is generally recognized that work fatigue is a condition of psychological and physiological decline caused by fatigue at work. Work fatigue can result from various factors, including prolonged continuous work, disruption of the internal clock, poor working conditions, insufficient sleep at night, inadequate rest breaks, excessive psychological pressure, and other unhealthy work practices [3]. Additionally, work fatigue is also related to the distribution of tasks; when participants are overloaded with work, the rate of fatigue accumulation increases [4]. Fatigue can reduce the ability of personnel and lead to adverse situations and even accidents, resulting in serious consequences [5].

Working speed is influenced by fatigue resulting from continuous work. To ensure normal operations and safeguard the safety of workers, it is necessary to provide appropriate compensation time. Given the varying individual characteristics of workers, as well as differences in working environments and the nature of tasks, the formulation of allowance time should be differentiated. Existing standards for allowance time often fail to be fully applicable. In the manufacturing industry, different standards for setting allowance time are prevalent, and are frequently either too high or too low [6]. Many enterprises lack a sufficient understanding of the importance of labor quotas, which can hinder the accurate setting of allowance time [7]. By establishing reasonable working hours through labor quotas, enterprises can maximize profits while minimizing the consumption of human, material, and other resources [8]. The formulation of allowance time can effectively mitigate the fatigue induced by operations; however, the methods for determining fatigue relaxation values remain underdeveloped [9]. In the absence of established relief time, workers are more prone to fatigue, which subsequently slows down productivity [10]. Various methods have been employed to develop appropriate fatigue allowances, such as the technical determination method, which can derive the corresponding broadening rate from actual work conditions [11]. Additionally, the relative metabolic rate (RMR) can be calculated to obtain the actual labor index for different workloads, facilitating the calculation of the corresponding fatigue slack rate [12]. Therefore, fatigue allowance holds significant importance within labor quotas in enterprises, and the reasonable formulation of this allowance is crucial for the scientific integrity of labor quotas. Moreover, while fatigue allowance is a necessary measure for enterprises to achieve optimal benefits, a comprehensive formulation method and standard are still lacking.

To address the aforementioned issues, this study breaks down machining production operations into common actions and simulates these actions in a laboratory setting. By collecting electromyography (EMG) and electrocardiography (ECG) signals through physiological measurements, the study examines how fatigue varies with different actions and varying continuous operating hours. Subsequently, a fatigue allowance rate model is established to provide fatigue allowances for various machining methods. The model of fatigue relaxation rate can provide a basis for fatigue allowances for different working methods of machining.

### 1.1. Theoretical Basis of Fatigue Allowance Measurement

**Figure 1:**
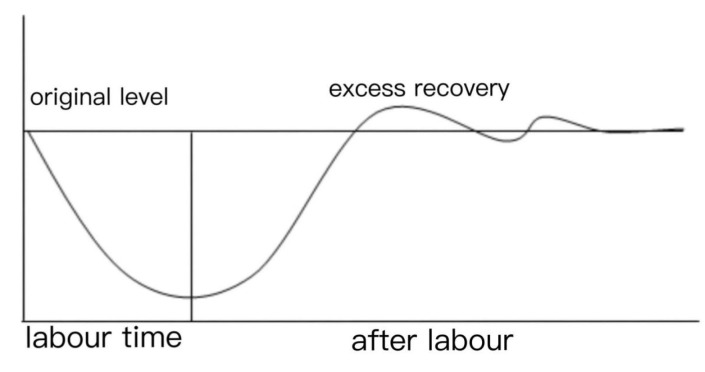
Diagram of ‘excess recovery’.

When the energy consumption associated with labor exceeds the body’s recovery capacity, the body depletes its energy reserves, leading to an accumulation of fatigue that must be replenished through rest [13]. Workers can achieve improved working conditions following adequate rest. During active work, the rate of energy consumption surpasses the rate of recovery, resulting in a decline in the body’s work capacity. Conversely, during rest, the recovery rate of energy substances exceeds the consumption rate, allowing the body’s energy levels and work capacity to gradually return to baseline. As recovery continues, the energy levels may temporarily surpass baseline levels before gradually declining. This phenomenon is referred to as ‘excess recovery’ or ‘excess compensation’ [14], as illustrated in Figure 1.

Following the phenomenon of ‘excess recovery’, if rest continues, energy levels will gradually return to baseline. Fatigue allowances should be designed to facilitate reaching the ‘excess recovery’ point, enabling staff to enhance their efficiency after rest.

### 1.2. Feasibility of the Physiological Measurement Method for Fatigue

The physiological measurement method can be employed to assess fatigue by measuring human physiological signals with specialized instruments, treating these signals as smooth random processes to reflect the real-time physiological and psychological state of the body. Some researchers have found a low crossover between fatigue assessment scales and have proposed that it is more appropriate to use physiological measurements to verify fatigue before determining fatigue allowances. The feasibility of physiological measurements in fatigue research has been highlighted previously [15]. Numerous studies have utilized physiological measurements to investigate the processes of fatigue onset and recovery. For instance, research has been conducted on fatigue generation and recovery through oxygen uptake and heart rate [16], muscle fatigue monitoring using electromyographic signals [17], analysis of fatigue trends via subject blink rates [18], and detection of user fatigue based on indicators such as blink duration, amplitude, and interval from eye movement signals [19], and research has also been carried out on advanced EEG methodologies for detecting driver fatigue [20] and to capture vibrations caused by physiological parameters such as pulse waves, heart sounds, and respiration through MEMS-based sensors [21]. By analyzing changes in physiological parameters, physiological indicators can be derived, allowing for the establishment of a relationship model between these indicators and time. These physiological indicators can then be utilized to evaluate human fatigue responses, providing an objective and quantitative measure of fatigue.

In this study, EMG and ECG were selected as physiological measures to reflect the degree of physiology, among which EMG can indicate the degree of muscle fatigue through RMS metrics [22,23,24] and ECG can indicate overall body fatigue through changes in HR metrics [25,26,27].

## 2. Materials and Methods

### 2.1. Experimental Environment and Equipment

**Figure 2:**
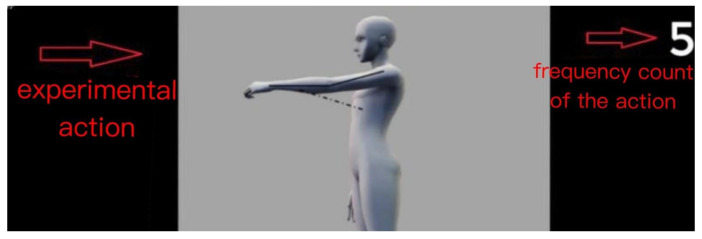
Experiment instruction video.

The experiment was conducted in the Human Factors Engineering Laboratory of the School of Economics and Management at Beijing Information Science and Technology University, simulating a production environment with a temperature range of 24–26 °C, adequate lighting, and ambient production noise. The production noise was derived from recordings of the actual production environment during the investigation. The experimental setup utilized the Kingfar Technology ErgoLAB human–computer environment synchronization platform, incorporating ErgoLAB 3.0 software along with EMG and ECG sensors. To ensure that subjects were familiar with the action process and to minimize artificial interference, a guidance video was used as the stimulus material, featuring a simulated demonstration of the experimental actions and the frequency counts of these actions, as shown in Figure 2.

### 2.2. Experiment Overview

To investigate the degree of fatigue caused by different movements and the rest time required for fatigue recovery under varying continuous operation durations, two experiments were designed in this study. Experiment 1 involved a fatigue measurement of actions at different frequencies, where subjects were instructed to perform action units at specified frequencies, followed by a period of fatigue recovery and rest. The degree of fatigue induced by the action units was assessed through linear changes in physiological indicators. Experiment 2 involved fatigue measurement during tasks of varying durations. Subjects combined action units in a rational manner to simulate machining operations, completing task simulations for 10, 20, and 40 min, respectively. Following these simulations, a fatigue recovery rest period was implemented. The required rest time was determined by analyzing changes in physiological indicators during the recovery phase under different continuous operation durations.

### 2.3. Decomposition of Machining Action Units

In this study, mechanical processing is selected as the research object. The machining operation consists of a series of processes that can be divided into combinations of several actions. Muscle fatigue is influenced by the intensity, rate, and duration of labor [28], meaning that the establishment of the fatigue allowance rate is affected by labor intensity, action rate, and continuous working hours. Referring to the National Occupational Health Standard of the People’s Republic of China (GBZ 2.2-2007) [29] and International Labor Organization (ILO) labor intensity classification standards, the labor intensity of mechanical processing belongs to class Ⅱ labor (medium labor). According to the survey and video extraction, the work contains steps for tool changing, installation, operation, inspection, unloading, transportation, cleaning, and other steps, as shown in Table 1.

The work in mechanical processing is mainly completed using the arms. Additionally, based on human factors engineering experiments, the Modapts system categorizes actions into 21 types to facilitate time measurement, most of which involve arm movements. Building on this, the above actions are further summarized into six action units, which are rotation, delivery, pushing, pressing down, holding, and holding and releasing (picking up/putting back), and their action frequencies were obtained, as shown in Table 2.

### 2.4. Experiment Subjects

The subjects should meet the requirements of mechanical workers, have the ability to engage in grade Ⅱ manual labor, be aged between 18 and 45 years old, have normal hearing and vision, have no experimental instruction perception impairment, have a body mass index (BMI) between 20 and 35, have no “underweight” or “very obese” phenomena, and have no disabilities or major diseases. A total of five participants were recruited for the experiment, and their information is presented in Table 3.

### 2.5. Experimental Procedure

**Figure 3:**
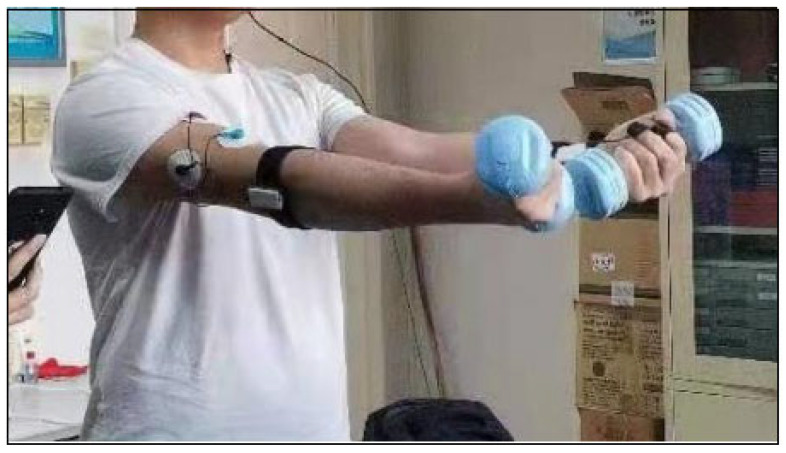
Experimental action attempt.

Before the experiment, the subjects were given a simple physical fitness test to ensure that they would not experience physical discomfort. After the sensor had been installed, the experimental subjects needed to listen to the action explanation and try the experimental actions to ensure the stability of physiological signal collection, as shown in Figure 3.

In Experiment 1, the actions of delivering, pushing, pressing down, rotating, and placing were performed successively at frequencies of 1, 3, 5, 10, 15, and 20 times. Additionally, the holding action was performed at frequencies of 1, 3, 5, 7, 10, and 12 times. To ensure that excessive recovery from fatigue did not affect the experiment, a 30 s rest was required between movements at different frequencies.

In Experiment 2, to recreate the machining site operation, a simulation of the site operation was developed according to the statistical ratio of the movements in machining and the content of the operation movements in different steps. According to the study in [30,31,32], the ratio of 8 in the machining of different actions was as follows: delivery:downward pressure:pick up:put down:rotation:push:hold = 10:20:10:10:20:5:1.

The formulated action combination was as follows: (1) Installation 15 s: pick up, deliver, put down, pick up, deliver, press down 5 times, and put down. (2) Tool change 20 s: pick up, deliver, press down 3 times, put down, pick up, deliver, press down 3 times, and put down. (3) Operation 40 s: pick up, deliver, rotate 5 times, press down 3 times, put down, pick up, deliver, rotate 10 times, rotate 5 times, and put down. (4) Inspection 10 s: pick up, deliver, measure (reading), and put down. (5) Unloading 20 s: pick up, deliver, press down 5 times, put down, pick up, deliver, and put down. (6) Transport 30 s: pick up, hold, and put down. (7) Clean up 15 s: torso forward, pushing 5 times, and torso recovery.

Experiment 2 was conducted in three groups, with 4, 8, and 16 repetitions of the action combinations, corresponding to 10 min, 20 min, and 40 min work simulation experiments, respectively. Each subject was required to perform the work simulation experiments for durations of 10, 20, and 40 min.

## 3. Results

### 3.1. EMG Indicators for Action Fatigue Experiments

**Figure 4:**
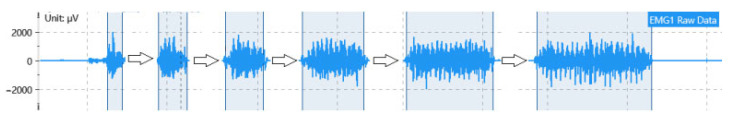
EMG signal of the delivery action at frequencies of 1/3/5/10/15/20.

Using the sEMG sensor, EMG signals generated by actions performed at different frequencies can be collected. For example, the collected signals for Subject 1 are shown in Figure 4.

To calculate the RMS index, the EMG signal is processed through filtering to obtain the signal data. The cutoff frequency for high-pass filtering is set to 5 Hz, the cutoff frequency for band-stop filtering is set to 50 Hz, and the cutoff frequency for low-pass filtering is set to 500 Hz. Following this, normalization and rectification using the sliding root mean square method are performed, with a maximum voluntary contraction (MVC) threshold of 1000 µV, an activation threshold of 10%, a minimum period of 1000 ms, and a minimum interval of 1000 ms to yield the EMG signal data. When the frequency is set to 1, the recording time is too short to analyze the signal data effectively. By substituting the valid EMG signal data and time *t* into Equation (1), the RMS index can be obtained.

(1) $$RMS=\sqrt{\frac{1}{T\int_{0}^{T}{EMG}^{2}(t)dt}}$$

where *EMG* represents the amplitude of the EMG signal and *T* represents the time at which the signal occurs.

**Figure 5:**
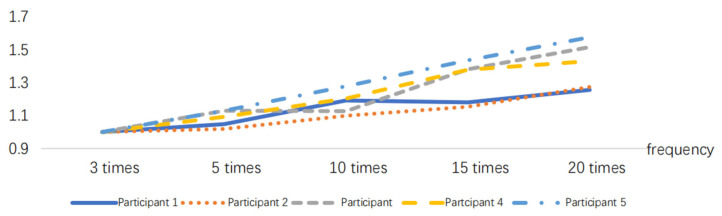
RMS variations in the delivery action at different frequencies.

**Figure 6:**
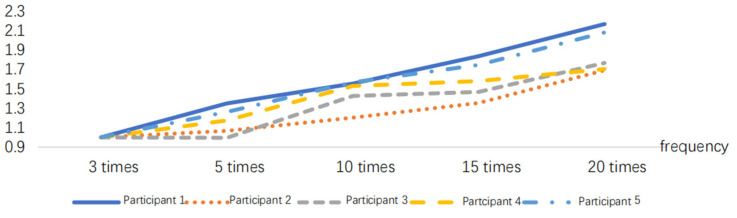
RMS variations in the pushing action at different frequencies.

**Figure 7:**
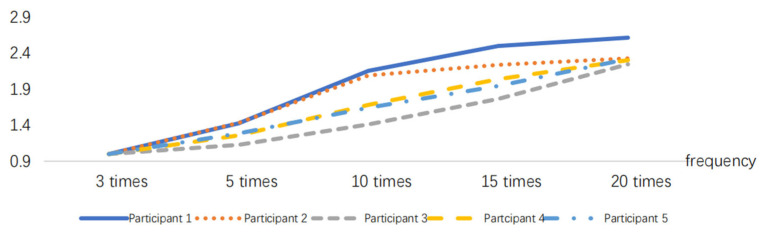
RMS variations in the pressing down action at different frequencies.

**Figure 8:**
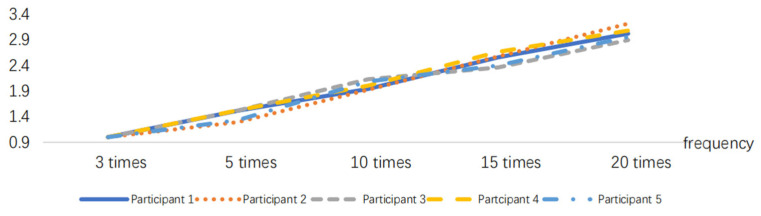
RMS variations in the rotating action at different frequencies.

**Figure 9:**
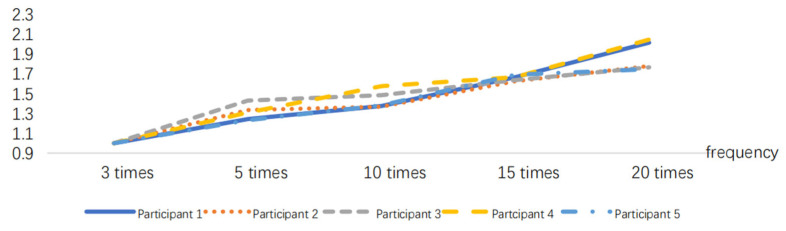
RMS variations in the placing action at different frequencies.

**Figure 10:**
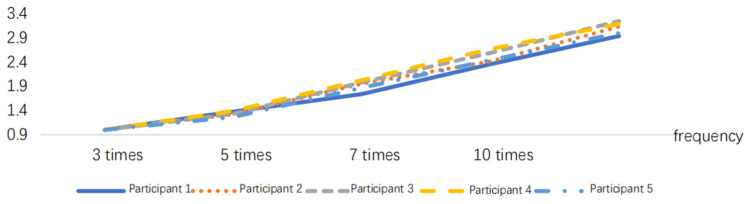
RMS variations in the holding action at different time durations.

After normalizing the obtained RMS index, the results are presented in Figure 5, Figure 6, Figure 7, Figure 8, Figure 9 and Figure 10.

### 3.2. EMG Indicators for Time-Lapse Fatigue Measurement Experiments

**Figure 11:**
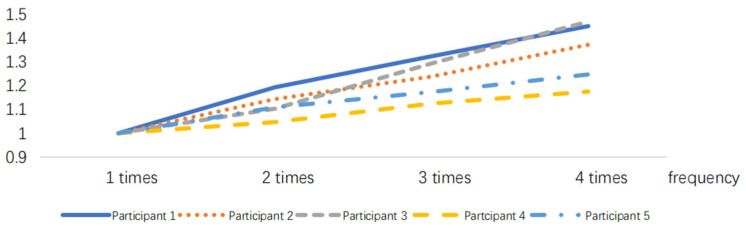
RMS variations in the 10 min task simulation experiment.

**Figure 12:**
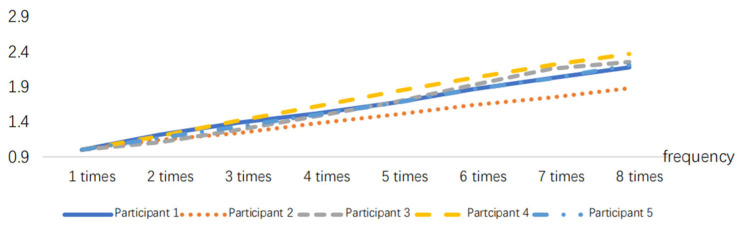
RMS variations in the 20 min task simulation experiment.

**Figure 13:**
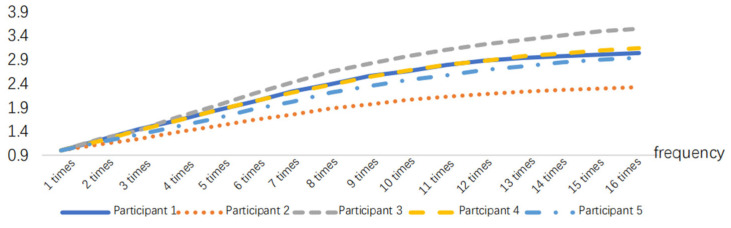
RMS variations in the 40 min task simulation experiment.

The EMG signals generated by the muscles during the simulated operation can be collected using sEMG sensors. The EMG signal data are processed as described in Section 3.1 to obtain the EMG signal. The RMS index can be calculated using the EMG signal data and the time *t*. To eliminate individual differences, the RMS values are normalized, as shown in Figure 11, Figure 12 and Figure 13.

**Figure 14:**
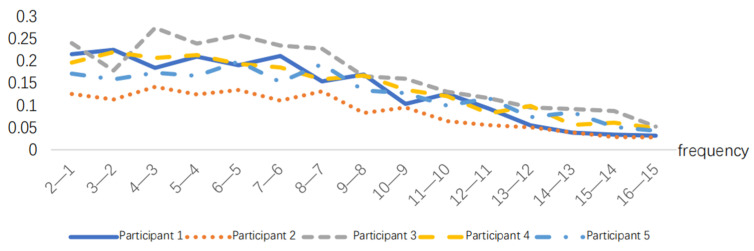
Slope between RMS points during the 40 min task experiment.

According to Figure 13, RMS presents a monotonically increasing trend, and the increasing trend will slow down when RMS increases to a certain threshold. The tendency for an increase in the time domain index slows down when the muscle available for recruitment decreases at the time of approaching fatigue, and the contraction synchronization space of the muscle fibers decreases and becomes smaller [33], that is, the fatigue destabilization phenomenon occurs. The experimenter observed that the subjects generally began to experience a decline in strength at the ninth movement combination, in aspects such as slower movement speed and reduced movement amplitude, leading to a decrease in muscle strength. The RMS curves of the 40 min work simulation experiment showed a decreasing trend in the slope between points from the eighth to the ninth time, i.e., the muscle strength was impaired, which is the same as the observed results, as shown in Figure 14.

### 3.3. ECG Metric Analysis for Time-Lapse Fatigue Measurement Experiments

The EMG signals generated by the heart during the simulated operation were collected using ECG sensors. When there was no significant difference between the heart rate (HR) index at a certain time and that during a resting state, this indicated a return to resting levels; this time was recorded as the fatigue recovery time [26]. To calculate the HR indicators, the ECG data were filtered using a high-pass cutoff frequency of 1 Hz, a band-stop cutoff frequency of 50 Hz, and a low-pass cutoff frequency of 100 Hz. The maximum heart rate was set at 120 bpm, the R-Peak threshold was established at 70%, and ectopic beats were detected and corrected based on the median. The HR indicators analyzed from the ECG signal received by the sensor were recorded approximately every 0.06 s. The HR data were intercepted at 1 s intervals, while subsequent data were intercepted at 0.1 s intervals. By comparing the differences in HR indicators between the resting period and the calm state, it was possible to determine whether the body had regained calmness [26]. The median of these time points was calculated and rounded to establish the completion time of rest. Due to the large volume of data in the HR index analysis, the interception interval was set to 5 s. The HR index was then imported into Statistical Product and Service Solutions (SPSS) version 24 for analysis, where a paired-sample *t*-test was conducted between the HR index during rest and the HR index during the pre-experimental quiet state. A significance level of *p* > 0.05 indicated no significant difference in the data, confirming that the body returned to resting levels at that time and that recovery from physiological fatigue had occurred. Thus, the resting time required for fatigue recovery was determined, as shown in Table 4.

### 3.4. Formulation of Fatigue Allowance

#### 3.4.1. Fatigue Allowance Rate Model Construction for Simulated Operations

From the analysis of the EMG index, it can be observed that the fatigue state gradually becomes unstable with increasing operational time. Therefore, the analysis should differentiate between the fatigue stability period and the fatigue instability period. Based on the RMS interpoint slope observed during the 40 min operation experiment, a significant decrease in the interpoint slope was noted between the eighth and ninth movement combinations, leading to the conclusion that the fatigue stabilization period is set at 20 min.

##### Fatigue Allowed Rate Model for Fatigue Stabilization Period

During the fatigue stabilization period, the RMS metric exhibited a linear relationship, indicating that the increase in fatigue level was linear. The rest time *T*_1_ and operational time *t* during this period were input into SPSS 24 for regression analysis, resulting in a rest time model. The coefficient of determination *R*^2^ was 0.991, indicating a good model fit and strong linearity, which reflects the regression model’s ability to effectively explain the variance of the dependent variable. The ANOVA significance value of *p* = 0.000 < 0.01 < 0.05 demonstrates that the linear regression model established with the dependent variable ‘rest time *T*_1_’ and the independent variable ‘work time *t’* is highly statistically significant. The model expression was derived from the unstandardized coefficients, as follows:

(2) $$T_{1}=0.07t-14.3$$

where *T*_1_ represents the rest time (in seconds) and *t* denotes the operating time (in seconds), constrained by 204.3 < *t* ≤ 1200.

The significance value of the regression coefficient for the independent variable ‘operating time’ is *p* = 0.000 < 0.01 < 0.05, indicating that the regression coefficient *b* is statistically significant. Thus, the regression coefficient passes the significance test, confirming a highly significant relationship. The formula for the fatigue relaxation rate during the fatigue stabilization period of the simulated operation is as follows:

(3) $$Y_{1}=0.07-14.3/t$$

where *Y*_1_ represents the fatigue relaxation rate during the fatigue stabilization period, and *t* denotes the operating time (in seconds), constrained by 204.3 < *t* ≤ 1200.

##### Fatigue Allowance Rate Model for the Fatigue Destabilization Period

During the fatigue destabilization period, muscle strength activation is inhibited, leading to a deceleration in the growth of the RMS index, while the fatigue level continues to accumulate, necessitating additional rest time for recovery. The fatigue index is defined as *G_n_ = RMS*_1_/ *RMS_n_ − RMS*_*n*−1_, where *RMS_n_* represents the RMS data of the *n*-th action combination. By substituting *n* values from 8 to 16, the fatigue index for the fatigue destabilization period was determined, and a fitting model was derived by inputting these values into SPSS 24. The curve fitting yielded a correlation coefficient of *R* = 0.856 > 0.85, indicating a good model fit and a robust linear equation. The significance value of *p* = 0.000 < 0.01 < 0.05 confirms that the fitted model, with the dependent variable ‘fatigue index *G’* and the independent variable ‘number of action combinations *n’*, is statistically significant. By using the unstandardized coefficients and substituting *n = t*/150, the model expression is derived as follows:

(4) $$G=18.762-3.456t/150+{0.24(t/150)}^{2}$$

where *G* is the fatigue index and *t* is the operating time (seconds), with the condition *t* ≥ 1200.

By substituting *t* = 1200/2400 into *G*, the fatigue index for the 20/40 min work destabilization period can be derived. The destabilization period fatigue index *G* and the corresponding rest time *T*_2_ are then input into SPSS 24 for regression fitting to establish the rest time model. The coefficient of determination *R*^2^ for this model was 0.983, indicating a good model fit and a strong linear relationship, thereby demonstrating the model’s capability to effectively explain the variance of the dependent variable. The ANOVA significance value of *p* = 0.000 < 0.01 < 0.05 suggests that the linear regression model linking the dependent variable ‘rest time *T*_2_’ and the independent variable ‘fatigue index *G’* is statistically significant. The unstandardized coefficients yielded the following model expressions:

(5) $$T_{2}=7.34G+21.978$$

The regression coefficient significance value of the independent variable ‘fatigue index *G’* is *p* = 0.000 < 0.01 < 0.05, indicating that the regression coefficient *b* is statistically significant. This confirms that the regression coefficient passes the test and demonstrates a highly significant relationship. The formula for the fatigue width expansion rate during the simulated fatigue instability period of operation is as follows:

(6) $$Y_{2}=7.34G/t+21.978/t$$

where *Y*_2_ is the fatigue width rate in the fatigue instability period, *G* is the fatigue coefficient, and *t* is the operation time (seconds), where *t* > 1200.

#### 3.4.2. Fatigue Coefficient of Different Movements

Fatigue induced by different actions can be assessed through the RMS index, which reflects linear fatigue performance. In this study, the action frequency is denoted as *a*, and the normalized RMS data for movement fatigue measurement is labeled as *b*. In the fatigue measurement experiments, various actions such as delivery, pushing, pressing down, rotating, holding, and releasing were assessed. The independent variable *a* and the dependent variable *b* were input into SPSS 24 for fitting analysis. The fitting models produced significant results, with all six models yielding significance values of *p* = 0.000 < 0.05. The slope coefficient of each fitting model represents the degree of fatigue associated with each action unit. If the fatigue coefficients of the different actions are represented as *f_n_*, then the ratios of the fatigue coefficients for delivery, pushing, pressing down, rotating, holding, and releasing are as follows: *f*_1_:*f*_2_:*f*_3_:*f*_4_:*f*_5_:*f*_6_ = 0.023:0.049:0.078:0.115:0.046:0.231.

#### 3.4.3. Fatigue Allowance Rate Model Based on Simulated Operations

Different operations involve varying numbers of action units, and the fatigue coefficient associated with each operation can be derived from the proportion of different actions performed. Let the fatigue coefficient for different operations be denoted as *F*. Therefore, *F* can be expressed as follows:

(7) $$F=\left(f_{1}x_{1}+f_{2}x_{2}+f_{3}x_{3}+f_{4}x_{4}+f_{5}x_{5}+f_{6}x_{6}\right)/\left(x_{1}+x_{2}+x_{3}+x_{4}+x_{5}+x_{6}\right)$$

Here, “*x*_1_, *x*_2_, *x*_3_, *x*_4_, *x*_5_, *x*_6_” are the number of actions of delivery, pushing, pressing, rotating, holding, and releasing.

In the designed simulation operation mode, the number of actions of delivery, pushing, pressing, rotating, holding, and releasing are 10, 5, 20, 20, 10, and 1 in order, and the fatigue coefficient of simulated operation *F*_0_ = (0.023*x*_1_ + 0.049*x*_2_ + 0.078*x*_3_ + 0.115*x*_4_ + 0.046*x*_5_ + 0.231*x*_6_)/(*x*_1_ + *x*_2_ + *x*_3_ + *x*_4_ + *x*_5_ + *x*_6_) = 0.076152. Depending on the ratio of the action, the fatigue allowance of other operations can be found based on the simulated mode of operation, and the fatigue allowance rates of different operations are set as follows:

(8) $$Y=F/F_{0}Y_{n}(t)$$

where *Y* is the fatigue allowance ratio, *Y_n_* is the fatigue allowance ratio of the simulation operation, *F* is the fatigue coefficient of the simulation operation, and *F*_0_ is the fatigue coefficient of the simulation operation. When 205.3 < *t* ≤ 1200, *n* = 1; when *t* > 1200, *n* = 2.

## 4. Discussion

This study focuses on a single processing method, deriving a fatigue tolerance model based on action decomposition, which can be extended to other processing methods. Future research could apply this approach to examine actions across diverse processing methods, contributing to the development of a more comprehensive fatigue recovery model. Additionally, the model can be corrected by updating the data in the future. The experimental subjects in this study were school students, and the fatigue relief rate model established for the simulated operation scheme showed good correspondence with the existing ILO time relief standards, but further corrections could be made using the physiological index data of the workers at the operation site to make the model more complete and accurate.

## 5. Conclusions

The reasonable formulation of the fatigue allowance rate is of great importance for both companies and employees. Through research on machining, a representative industry in the manufacturing industry, six action units were obtained and combined to simulate machining operations, and the simulated operations with different frequencies of action units and different time durations were studied by using physiological measurements. The study used the RMS index fitting model of the action fatigue measurement experiment to obtain the ratio of fatigue caused by action units, obtained the rest time for fatigue recovery by analyzing the HR index, obtained the fatigue destabilization time point by using the RMS index data of the operation fatigue measurement experiment, and then obtained the fatigue allowance rate model of the simulated operation method by using the continuous operation length and the corresponding fatigue allowance rate. The number of action units in different processing methods is different, and the fatigue allowance rate can be calculated for different continuous operating hours in different processing methods by using the proportion of action units in different processing methods.

## Figures and Tables

**Table 1:** Work content of machining.

Steps	Job Description	Primary Mode of Action
Tool change	Manual: Put the tool into the tool holder, rotate the handle of the tool holder to close tight clamp	Single-arm operation
	Automatic: operate CNC machine tools to achieve automatic tool change	Key press operation
Installation	Put the workpiece into the spindle box, and use a tool such as a hexagonal wrench to tighten the fixture with downward pressure.	Single-arm operation
Operation	Feed: rotate the handle at the skid plate into the tool, observe the workpiece processing	Single-arm operation
	Automatic feed: realize feed through the button and observe the workpiece processing	Key press operation
Inspection	Use of measuring tools to verify that the workpiece is machined as required	Single-arm operation
Discharge	To remove the workpiece from the fixture, use a tool such as a hexagonal wrench	Single-arm operation
Shipping	Freehand handling of finished workpieces	Two-arm operation
Clearance	Clean the iron filings from the countertop and tools, clean them to the ground and push and sweep them to the fixed area to wait for centralized cleaning	Single-arm operation

**Table 2:** Action units and single group time standards.

Movement Unit	Movement Standard	Single Time	Single Standard
Rotation	The dominant arm is parallel to the ground, with the elbow as axis; the non-dominant arm conducts the rotation	2 s/lap	The dominant arm does not move, and the non-dominant arm rotates one turn as a complete action
Delivery	Arms parallel to the ground; retract and straighten	2 s/set	A complete set of movements with one arm bent back and straight out
Push	The load is placed on a flat surface, and the arm holds the negative load; do retract and straighten	2 s/time	Bending the arms to retract and straighten them to send out for a complete set of movements
Downward pressure	Non-dominant arm and dominant arm in a straight line, flat up and down, amplitude 20~45°	2 s/set	A complete set of movements with a single up-and-down lift of the dominant arm
Hold	Dominant arm down, dominant arm at 90° to non-dominant arm angle, holding the load with both hands	15 s/time	Hold the load for 15 s and release it as a complete action
Take and place	Dominant arm does not move, palm down, move the non-dominant arm in the target position to grab/place the load	2 s/time	Pick up/put back small parts at the specified target point as a complete action

**Table 3:** Participant information.

Participant	Gender	Age	BMI	Disability/Serious Illness	Experience in Grade Ⅱ Manual Labor
1	Male	24 years	23.6	No	Yes
2	Male	27 years	27.1	No	Yes
3	Male	26 years	25.6	No	Yes
4	Male	24 years	25.4	No	Yes
5	Male	28 years	27.7	No	Yes

**Table 4:** Fatigue recovery rest time for the 10 min operation experiment (unit: s).

Subject	10 min Assignment Experiment		20 min Assignment Experiment		40 min Assignment Experiment	
	Action Time	Rest Time	Action Time	Rest Time	Action Time	Rest Time
1	600	29	1200	73.5	2400	220
2	600	27.5	1200	67	2400	182.5
3	600	27	1200	69.5	2400	211.5
4	600	26	1200	66.5	2400	207
5	600	28.5	1200	71	2400	203

## Data Availability

The data is unavailable due to privacy.

## References

[1] The Time Measurement Method of Labor Quota.

[2] Noy Y.I., Horrey W.J., Popkin S.M., Folkard S., Howarth H.D., Courtney T.K. (2011). Future directions in fatigue and safety research. Accid. Anal. Prev..

[3] Tourangeau A.E., Cranley L.A. (2010). Nurse intention to remain employed: Understanding and strengthening determinants. J. Adv. Nurs..

[4] Ahsberg E. (2010). Dimensions of fatigue in different working populations. Scand. J. Psychol..

[5] Song W.S. (2015). Study on Pilots’ Fatigue Risk Management. Master’s Thesis.

[6] Mital A., Bishu R.R., Manjunath S.G. (1991). Review and evaluation of techniques for determining fatigue allowances. Int. J. Ind. Ergon..

[7] Liu Y.M. (2002). Several problems of labor quota work in stateIn Proceedings of the-owned enterprises. China Labor..

[8] Tang X.G., Xu H.J., Cai Q.M. (2004). Analysis and formulation of labor quota in industrial enterprises. Commer. Res..

[9] Ren J. (2013). The Formulation of Work Quota A Parts Warehouse Workers. Master’s Thesis.

[10] Yan Y.H., Wang F. (2016). The application of model timing method in the improvement of labor quota. Technol. Innov. Product..

[11] Zhu H.Z., Wu L.F. (2020). Exploration and practice of labor quota management system in Liangshuijing Coal Mine. Enterp. Reform. Manag..

[12] Zhou J.Q., Wang H.Y., Yu Z.Q., Li Y. (2021). Quantitative evaluation of human engineering for selection of logistics distribution center. Logist. Technol..

[13] Guo F., Qian S.S. (2018). Human Factors Engineering.

[14] Zhang D.Y. (1993). Research on the Allowance Time.

[15] Lund J., Mericle K.S. (2000). Determining fatigue allowances for grocery order selectors. Appl. Ergon..

[16] Hsin-Chienh W.U., Hsu W.H., Chen T. (2005). Complete recovery time after exhaustion in high-intensity work. Ergonomics.

[17] Alzaman A., Ferdjallah M., Khamayseh A. Muscle Fatigue Analysis for Healthy Adults Using TVAR Model with Instantaneous Frequency Estimation. Proceedings of the 2006 Proceeding of the Thirty-Eighth Southeastern Symposium on System Theory.

[18] Zeng Y.W., Feng Z., Zhu Y.B., Li Q. (2017). Correlation between blinking frequency and fatigue based on EEG experiment. J. Chang Univ. Sci. Technol..

[19] Kolodziej M., Tarnowski P., Sawicki D.J., Majkowski A., Rak R.J., Bala A., Pluta A. (2020). Fatigue Detection Caused by Office Work with the Use of EOG Signal. IEEE Sens. J..

[20] Hu F., Zhang L., Yang X., Zhang W.-A. (2024). EEG-Based Driver Fatigue Detection Using Spatio-Temporal Fusion Network With Brain Region Partitioning Strategy. IEEE Trans. Intell. Transp. Syst..

[21] Nguyen T.-V., Okada H., Takei Y., Takei A., Ichiki M. A Band-Aid Type Sensor for Wearable Physiological Monitoring. Proceedings of the 2021 21st International Conference on Solid-State Sensors, Actuators and Microsystems (Transducers).

[22] Ma J.Y. (2015). Study on Surface EMG Analysis Methods Under Rehabilitation Exercise. Master’s Thesis.

[23] Venugopal G., Nacaneethakrishna M., Ramakrishnan S. (2014). Extraction and analysis of multiple time window features associated with muscle fatigue conditions using sEMG signals. Expert Syst. Appl..

[24] Al Harrach M., Carriou V., Boudaoud S., Laforet J., Marin F. (2017). Analysis of the sEMG/force relationship using HD-sEMG technique and data fusion: A simulation study. Comput. Biol. Med..

[25] Song T., Yuan C., Wu Y., Li Y., Wu P. (2017). Research on the application of HRV in the experimental teaching of exercise fatigue judgment. Lab. Sci..

[26] Wang Y.H., Qi C.H., Zhu S.L., Xie S.F., Zhao T. (2017). Ecg signal analysis of recovery time from driver fatigue. China Saf. Sci. J..

[27] Xu M.W., Jin L.Z., Zhang L., Yu L., Liu J.G., Tia X.H. (2018). Analysis of physiological fatigue of construction workers based on heart rate monitoring. Chin. J. Eng..

[28] Wang Y., Liu Y., Xu M.W. (2019). Experimental study on upper limb muscle fatigue of workers based on surface, E.M.G. J. North China Inst. Sci. Technol..

[29] Occupational exposure limits for hazardous agents in the workplace Part 2: Physical agents.

[30] Wang X.K. (2020). Machinery Technology.

[31] Li Y.M., Jin W.D. (2013). Machine Manufacturing Technology.

[32] Liu Y., Xu Y., Yang J. (2014). Fundamentals of Machining Technology.

[33] Bu Y.F., Li S.M. (2013). Nonlinear relationship between muscle sEMG and exercise load during the process of increasing load induced muscle fatigue. J. Shandong Sport Univ..

